# Heat Stress-Induced DNA Damage

**Published:** 2016

**Authors:** O.L. Kantidze, A.K. Velichko, A.V. Luzhin, S.V. Razin

**Affiliations:** Institute of Gene Biology, Russian Academy of Sciences, Vavilova str. 34/5, 119334, Moscow, Russia; Lomonosov Moscow State University, Faculty of Biology, Leninskie Gory 1, bldg. 12, 119991, Moscow, Russia

**Keywords:** heat shock, DNA repair, DNA damage, DNA replication, topoisomerase

## Abstract

Although the heat-stress response has been extensively studied for decades, very little is known about its effects on nucleic acids and nucleic acid-associated processes. This is due to the fact that the research has focused on the study of heat shock proteins and factors (HSPs and HSFs), their involvement in the regulation of transcription, protein homeostasis, etc. Recently, there has been some progress in the study of heat stress effects on DNA integrity. In this review, we summarize and discuss well-known and potential mechanisms of formation of various heat stress-induced DNA damage.

## INTRODUCTION

Heat stress (heat shock, hyperthermia) is one of the most well-studied complex stress factors. Cell response to heat stress involves most sub-cellular compartments and metabolic processes [1-3]. It has long been known that cells exposed to heat stress display an increased sensitivity to agents inducing double-stranded DNA breaks (DSBs), in particular to ionizing radiation [4, 5]. This phenomenon is called “heat radiosensitization.” It was assumed that this effect is caused by the fact that heat stress can inhibit the DNA repair system [5]. Indeed, several decades-long studies have shown that heat stress can inhibit the key components of virtually all repair systems *(Figure). *Heat stress inhibits the activity of the base excision repair (BER) system [6-9] and nucleotide excision repair (NER) system [10, 11]. The effect of heat stress on base excision repair has been the most extensively studied: heat stress can directly inactivate DNA polymerase β and certain DNA glycosylases [6, 9]. Recently, it has been shown that heat stress may also inhibit the mismatch repair system [12]. Inhibition of DSB repair systems resulting from heat stress makes the largest contribution to heat*-*induced radiosensitization. It is known that heat stress inhibits the functioning of both the non-homologous DNA end joining (NHEJ) system and the homologous recombination (HR) system. In the case of NHEJ, the effect of heat stress is limited by the complex of DNA-dependent protein kinase (DNA-PK): it was shown that hyperthermia can lead to aggregation of the Ku70/80 heterodimer (and therefore reduction in its DNA-binding activity), inhibition of Ku80 expression and/or inhibition of the DNA-PK catalytic subunit [13-15]. The situation is different with HR: heat stress may inhibit this repair system at several key stages [16]. The impact of hyperthermia on DNA repair systems in higher eukaryotes is discussed in the recently published review by P.M. Krawczyk *et al*. [17]; so we suggest that our readers consult this review, while our mini-review will mainly focus on direct heat stress-induced DNA damage *(Figure).*

**Figure:**
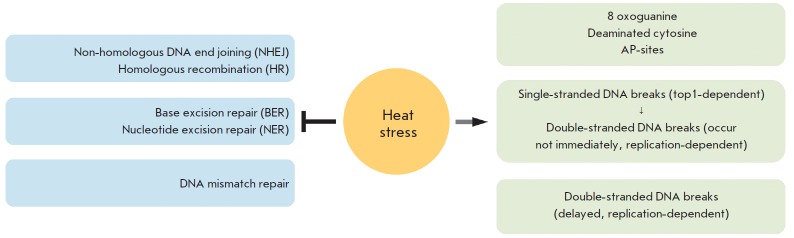
The effect of heat stress on the integrity of DNA and the repair system (see text for details)

**Single-stranded DNA breaks induced by heat stress**

Heat stress not only inhibits DNA repair systems, but can also act as a DNA damaging agent. It is known that heat stress can lead to the accumulation of 8-oxoguanine, deaminated cytosine, and apurinic DNA sites (AP-sites) in a cell [18-20]. It can be suggested that such DNA damage, as well as single-stranded DNA breaks (SSBs), is passively accumulated in the cell due to heat stress-induced inhibition of excision repair systems. A more interesting and controversial question is related to the nature of heat stress-induced DSBs, as well as the possibility of active heat stress induction of SSBs. For a long time, it was believed that heat stress does not induce DSBs, but rather leads to the generation of SSBs, which are formed as a result of inhibition of DNA replication due to hyperthermia [21-23]. We used several complementary approaches (comet assay, fluorescent *in situ *labeling of DNA breaks using DNA polymerase I) to demonstrate that heat stress, indeed, induces SSBs in cells during the S-phase of the cell cycle [24]. In the same paper, it was shown that hyperthermia can inhibit DNA replication: heat stress leads to either a slowing-down or arrest of replication forks, depending on the temperature and cell line [24]. However, it should be noted that the occurrence of SSBs in S-phase cells is not associated with heat stress-induced inhibition of DNA replication [25]. Recently, we have identified the mechanism of heat stress-induced SSBs. It was found that heat stress induces SSBs by inhibition of DNA topoisomerase I (top1), an enzyme that relaxes DNA supercoils by introducing temporary SSB into DNA [25]. The catalytic cycle of top1 includes cleavage of one DNA strand, accompanied by formation of an intermediate complex consisting of the enzyme covalently bound to the DNA. Stabilization of this complex is the main mechanism of genotoxic action of top1 poisons (e.g., camptothecin and its derivatives) [26, 27]. Heat stress (45°C) can not only inhibit the catalytic activity of the enzyme, but also lead to the accumulation of covalently bound top1-DNA complexes in the cell. It can be concluded that the effect of hyperthermia on top1 is similar to the action of poisons. The only difference is that heat stress is likely to suppress top1 activity at all stages of the catalytic cycle. Although it is known that top1 can bind to preexisting SSBs in the cell [28, 29], in the case of heat stress it is top1 that causes their emergence. The most convincing evidence of this was obtained in experiments with inhibition of enzyme expression through RNA interference [25]. It has been shown that, in the case of decreased expression of top1, the cellular senescence program, which depends on SSBs induction and their conversion into persistent DSBs, is not activated [25]. This is indicative of the fact that no heat stress-induced formation of SSBs occurs in cells not expressing top1. Therefore, the role of top1 in the formation of heat stress-induced SSBs seems to be quite obvious. It is also interesting that, in HeLa cells, covalently bound complexes between top1 and DNA are effectively formed only at temperatures above 44°C. Therefore, SSBs should not form at the clinically relevant temperatures of 41–43°C. Heat stress-induced formation of SSBs is mainly observed in the S-phase of the cell cycle, because the main function of top1 is to resolve topological problems that occur during DNA replication. We can state that the sensitivity of non-proliferating cells (terminally differentiated, arrested in G0 phase, etc.) should be significantly reduced in terms of the formation of SSBs. It cannot be completely absent, as the function of top1 in the cell is not limited to the DNA replication process. In this regard, it is worth noting that heat stress induction of SSBs is likely to occur not only in the S-phase of the cell cycle. SSBs also form in the G1 and G2 phases, but with very low frequency. According to our unpublished data, the number of SSBs formed due to heat stress in various cell lines directly correlates with the level of top1 expression. Summarizing these findings, we can conclude that heat stress inhibits the *in vivo *activity of top1 and leads to the formation of covalently bound complexes between the enzyme and the DNA and, as a consequence, formation of SSBs.

**Double-stranded DNA breaks induced by heat stress**

Heat stress-induced SSB is a source of DSB formation. These DSBs have several interesting features: they are specific to the S-phase of the cell cycle and occur in the cell not immediately after the heat stress, but rather 3–6 hours later [25]. These delayed DSBs occur due to the collision of replication forks which were re-started after heat stress-induced arrest, with SSBs, resulting from top1 inhibition [25]. Slow kinetics of the formation of these DSBs is associated with heat stress-induced inhibition of DNA replication, on the one hand, and inhibition of the transcription process, on the other hand [25]. Active transcription process is required for the detection and subsequent removal of the top1 complex covalently bound to DNA, resulting in SSB unmasking and the possibility of their collision with replication forks [30, 31]. Apparently, delayed DSBs are effectively recognized by cellular systems, as evidenced by ATM/ ATR-dependent phosphorylation of H2AX (DSB marker), followed by the involvement of other repair factors to the break site (53BP1, Rad51, etc.). However, repair of these breaks does not occur, which leads to the occurrence of a persistent DNA damage signal in the cell and, consequently, to initiation of a premature cellular senescence program [25, 32].

As can be seen from the abovementioned, we were the first to established the mechanism of delayed DSB formation under heat stress conditions. However, the question of whether heat stress can immediately induce DSB has long remained a controversial one. In recent years, it has been shown in different laboratories that heat stress can induce phosphorylation of H2AX histone [33-37], which is one of the first events in the processes of DSB recognition and repair [38, 39]. However, interpretation of these results is quite contradictory: some researchers have stated that γH2AX foci mask heat-induced DSBs [34, 37]; others believe that heat shock itself does not lead to DNA damage and, in this case, γH2AX is a byproduct of the cellular response to stress [33, 35, 36]. Recently, we have proved that hyperthermia can provoke the formation of DSBs [24, 40]. This was confirmed using two independent approaches: comet assay and labeling of DNA ends with terminal deoxynucleotidyl transferase. However, heat stress induces DSBs only in G1- and G2-phase cells. These DSBs are marked by ATM-dependent phosphorylation of H2AX [24]. Interestingly, other repair factors, such as the 53BP1 protein, are not attracted to γH2AX foci immediately after exposure to hyperthermia [24, 41]. At the same time, these DSBs are effectively repaired within the first 3—6 hours after heat stress. This probably means that active repair of heat stress-induced DSBs does not begin immediately after exposure, but rather some time after – when heat stress-inhibited repair systems have recovered. However, the mechanism (trigger) of immediate formation of DSBs under heat stress conditions is still not understood. The following processes can be considered as possible candidates for this role: activation of retroelements [42, 43], generation of reactive oxygen species [18], and transcription arrest [44, 45]. It is well-known that the aforementioned processes can lead to the formation of DSBs and, under certain conditions, occur during heat stress. However, none of these hypotheses can provide a convincing explanation of heat stress induction of DSBs in non-S-phase cells only. In our opinion, the most probable mechanism of heat stress-induced formation of DSBs is to inhibit the activity of DNA topoisomerase II (top2), an enzyme that changes DNA topology by introducing temporary DSBs [46]. Such discontinuities are accompanied by the formation of a covalent bond between the protein molecule and one end of the DNA chain. Inhibition of top2 at the stage of covalently bound complex leads to the formation of DSBs [46]. The results showing that heat stress can inhibit the activity of top2 *in vitro *were obtained long ago [47]. The fact that heat stress can reduce the genotoxic potential of top2 poisons is also indicative of the influence of hyperthermia on this enzyme [48]. There are two isoforms of top2, and expression of one of them depends on the stage of the cell cycle [49, 50]. This dynamics of expression could easily explain the dependence of DSB induction on the cell cycle phase.

## CONCLUSION

In summary, we can state that, in addition to complex suppression of almost all the repair systems in the cells of higher eukaryotes, heat stress directly results in the formation of various DNA damage. Interestingly, the type and the fate of the heat stress-induced damage depends on the stage of the cell cycle when the cell is exposed to high temperatures. For example, in the S phase of the cell cycle, hyperthermia leads to a top1-dependent formation of SSBs, some of which can be converted into difficult-to-repair DSBs several hours later. At the same time, heat stress immediately induces DSB formation in cells that are at the G1 or G2 stage of the cell cycle. Although this scheme of heat stress action is characteristic of all cell lines analyzed in our study, it should be kept in mind that the number of breaks and the degree of repair response of the cell can considerably vary depending on the strength of the heat stress and the cell type (line).

## Abbreviations

AND TERMS SSB: single-stranded DNA break
DSB: double-stranded DNA break
top1: DNA topoisomerase I
top2: DNA topoisomerase II
